# Oleuropein, a Component of Extra Virgin Olive Oil, Improves Liver Steatosis and Lobular Inflammation by Lipopolysaccharides–TLR4 Axis Downregulation

**DOI:** 10.3390/ijms25115580

**Published:** 2024-05-21

**Authors:** Leonardo Schirone, Diletta Overi, Guido Carpino, Roberto Carnevale, Elena De Falco, Cristina Nocella, Alessandra D’Amico, Simona Bartimoccia, Vittoria Cammisotto, Valentina Castellani, Giacomo Frati, Sebastiano Sciarretta, Eugenio Gaudio, Pasquale Pignatelli, Domenico Alvaro, Francesco Violi

**Affiliations:** 1 IRCCS Neuromed, 86077 Pozzilli, Italy; leonardo.schirone@uniroma1.it (L.S.); roberto.carnevale@uniroma1.it (R.C.); giacomo.frati@uniroma1.it (G.F.); sebastiano.sciarretta@uniroma1.it (S.S.); 2 Department of Anatomical, Histological, Forensic Medicine and Orthopedic Sciences, Sapienza University of Rome, 00185 Rome, Italy; diletta.overi@uniroma1.it (D.O.); guido.carpino@uniroma1.it (G.C.); eugenio.gaudio@uniroma1.it (E.G.); 3 Department of Medical-Surgical Sciences and Biotechnologies, Sapienza University of Rome, 04100 Latina, Italy; elena.defalco@uniroma1.it (E.D.F.); alessandra.damico@uniroma1.it (A.D.); simona.bartimoccia@uniroma1.it (S.B.); 4 Department of Clinical Internal, Anesthesiological and Cardiovascular Sciences, Sapienza University of Rome, 00185 Rome, Italy; cristina.nocella@uniroma1.it (C.N.); vittoria.cammisotto@uniroma1.it (V.C.); pasquale.pignatelli@uniroma1.it (P.P.); 5 Department of General Surgery and Surgical Speciality Paride Stefanini, Sapienza University of Rome, 00185 Rome, Italy; valentina.castellani@uniroma1.it; 6 Department of Precision and Translational Medicine, Sapienza University of Rome, 00185 Rome, Italy; domenico.alvaro@uniroma1.it

**Keywords:** gut dysbiosis, high-fat diet, liver steatosis, oleuropein, lipopolysaccharides

## Abstract

Gut-dysbiosis-induced lipopolysaccharides (LPS) translocation into systemic circulation has been suggested to be implicated in nonalcoholic fatty liver disease (NAFLD) pathogenesis. This study aimed to assess if oleuropein (OLE), a component of extra virgin olive oil, lowers high-fat-diet (HFD)-induced endotoxemia and, eventually, liver steatosis. An immunohistochemistry analysis of the intestine and liver was performed in (i) control mice (CTR; n = 15), (ii) high-fat-diet fed (HFD) mice (HFD; n = 16), and (iii) HFD mice treated with 6 µg/day of OLE for 30 days (HFD + OLE, n = 13). The HFD mice developed significant liver steatosis compared to the controls, an effect that was significantly reduced in the HFD + OLE-treated mice. The amount of hepatocyte LPS localization and the number of TLR4+ macrophages were higher in the HFD mice in the than controls and were lowered in the HFD + OLE-treated mice. The number of CD42b+ platelets was increased in the liver sinusoids of the HFD mice compared to the controls and decreased in the HFD + OLE-treated mice. Compared to the controls, the HFD-treated mice showed a high percentage of intestine PAS+ goblet cells, an increased length of intestinal crypts, LPS localization and TLR4+ expression, and occludin downregulation, an effect counteracted in the HFD + OLE-treated mice. The HFD-fed animals displayed increased systemic levels of LPS and zonulin, but they were reduced in the HFD + OLE-treated animals. It can be seen that OLE administration improves liver steatosis and inflammation in association with decreased LPS translocation into the systemic circulation, hepatocyte localization of LPS and TLR4 downregulation in HFD-induced mouse model of NAFLD.

## 1. Introduction

Nonalcoholic fatty liver disease (NAFLD) is characterized by the accumulation of fatty acids within hepatocytes, which may progress to nonalcoholic steatohepatitis (NASH), cirrhosis, and eventually liver cancer [1]. In the general population, the prevalence of NAFLD is 20–29%, and 20–30% of these cases may progress to NASH [2]. The mechanisms accounting for NAFLD are not entirely understood yet; however, evidence has shown that gut-derived endotoxemia may have a role in it, as suggested by the observation of lipopolysaccharides (LPS) localization within the liver cells of patients with NAFLD and NASH, and its association with activated, pro-inflammatory macrophages [3]. LPS constitutes the outer membrane of Gram-negative gut microbiota and may translocate into systemic circulation in the case of gut dysbiosis, which is characterized by changes in the composition of gut microbiota with a predominance of pathogenic bacteria [4]. Thus, experimental and clinical evidence suggests that gut dysbiosis may be implicated in the pathogenesis of NAFLD, with Escherichia coli being the most abundant bacterium, via the overproduction of LPS and ensuing endotoxemia [5,6,7]. This hypothesis is corroborated by experimental models showing that the injection of low-dose LPS results in fatty acid accumulation in the liver or steatohepatitis [8], with an attenuation of inflammation-mediated liver damage in animals with a loss of LPS-binding protein [9]. The biological validity of this hypothesis hinges on the premise that, upon entering the portal circulation, LPS is absorbed by hepatocytes and Kupffer cells. Subsequently, through interaction with Toll-like receptor 4 (TLR4), it actively promotes liver inflammation [2]. Concurrently, animals lacking TLR4 exhibit a reduced susceptibility to non-alcoholic fatty liver disease (NAFLD) [10]. According to this argument, it is tempting to speculate that modulation of gut dysbiosis-related endotoxemia could lower liver inflammation and, eventually, attenuate the features of NAFLD. Previous studies in which extra virgin olive oil (EVOO) was administered to animals given a high-fat diet demonstrated a positive effect by modulating gut dysbiosis, improving gut intestinal barrier permeability, and lowering LPS localization in the liver [11]. Similarly, human studies showed improved gut permeability and reduced endotoxemia in patients with diabetes treated with EVOO or its phenolic compound oleuropein (OLE) [12,13], which is known for its antioxidant, anti-inflammatory, and hepatoprotective properties. Studies have shown that oleuropein can attenuate liver steatosis induced by a high-fat diet in mice by modulating fat metabolism and reducing inflammatory responses in the liver [14,15]. This is supported by gene expression profiling, which identifies the downregulation of lipogenesis and inflammation pathways as mechanisms of action [16]. Additionally, oleuropein has shown protective effects against various liver injuries beyond steatosis. It has been effective in reducing oxidative stress and inflammation in models of liver damage, such as those induced by carbon tetrachloride [17] or cadmium [18]. This highlights its broad therapeutic potential for liver diseases. Based on these data, we tested the hypothesis that administering OLE to animals given a high-fat diet (a mouse model of NAFLD) could improve gut permeability, endotoxemia, and NAFLD features.

## 2. Results

### 2.1. Oleuropein Reduces HFD-Induced Increased Platelet Activation and Higher Concentrations of Circulating LPS

**Figure 1:**
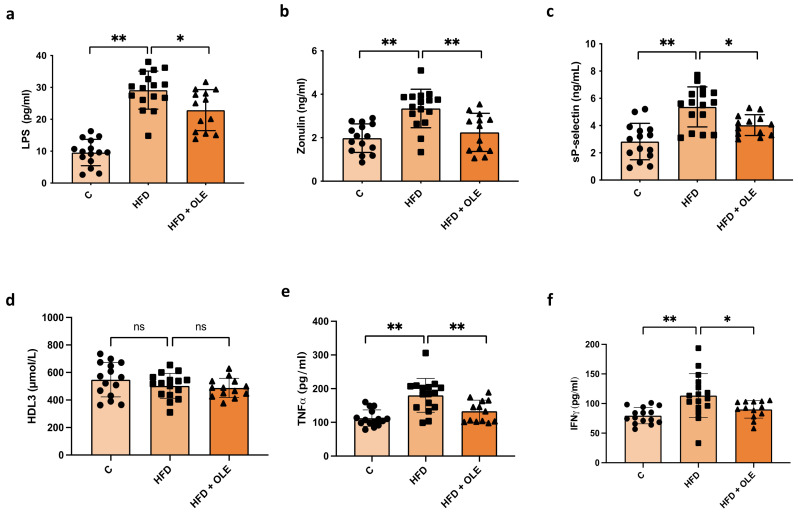
Gut permeability, platelet activation, and inflammation in mice fed a high-fat diet (HFD) and after oleuropein treatment. (**a**) Lipopolysaccharide (LPS), (**b**) zonulin, (**c**) sP-selectin, (**d**) HDL3, (**e**) TNF-α, and (**f**) IFN-γ in the serum of HFD mice compared to the control group and mice treated with oleuropein (HFD + OLE). * *p* < 0.05; ** *p* < 0.01; ns = not significant.

Wild-type mice were fed with a normal diet (C), a high-fat diet (HFD), or a customized HFD including OLE (HFD + OLE). The HFD-fed mice’s sera had a higher LPS and zonulin concentrations than the control group (Figure 1a,b). Moreover, mice given the HFD + OLE displayed decreased levels of LPS and zonulin compared to those given teh HFD alone (22.89 ± 6.42 vs. 29.15 ± 5.97 pg/mL, *p* < 0.05 and 2.24 ± 0.88 vs. 3.40 ± 1.023 ng/mL, *p* < 0.01, respectively). In addition, the sP-selectin concentration was increased in the HFD groups compared to the controls, suggesting high levels of platelet activation. Conversely, HFD mice treated with OLE showed a significant reduction in their sP-selectin levels compared to the HFD mice not supplemented with OLE (4.03 ± 0.76 vs. 5.37 ± 1.47 ng/mL, *p* < 0.05) (Figure 1c). Lastly, no differences were observed in serum HDL3 concentrations between the control diet and HFD groups, and between the HFD and HFD + OLE groups. (Figure 1d). Regarding inflammatory status, we observed that the HFD-fed mice’s sera had a higher TNF-α and IFN-γ concentrations than the control group (Figure 1e,f). Moreover, mice given a HFD + OLE displayed decreased levels of TNF-α and IFN-γ compared to HFD alone (133.2 ± 32.61 vs. 180.1 ± 50.26 pg/mL, *p* < 0.01 and 90.13 ± 14.72 vs. 113.40 ± 37.02 pg/mL, *p* < 0.05; respectively) (Figure 1e,f). A linear regression analysis demonstrated that LPS directly correlated with TNF-α (rS = 0.529, *p* < 0.01) and IFN-γ (rS = 0.523, *p <* 0.01).

### 2.2. Oleuropein Reduces HFD-Induced Hepatic Steatosis, Lobular Inflammation, and Fibrosis

**Figure 2:**
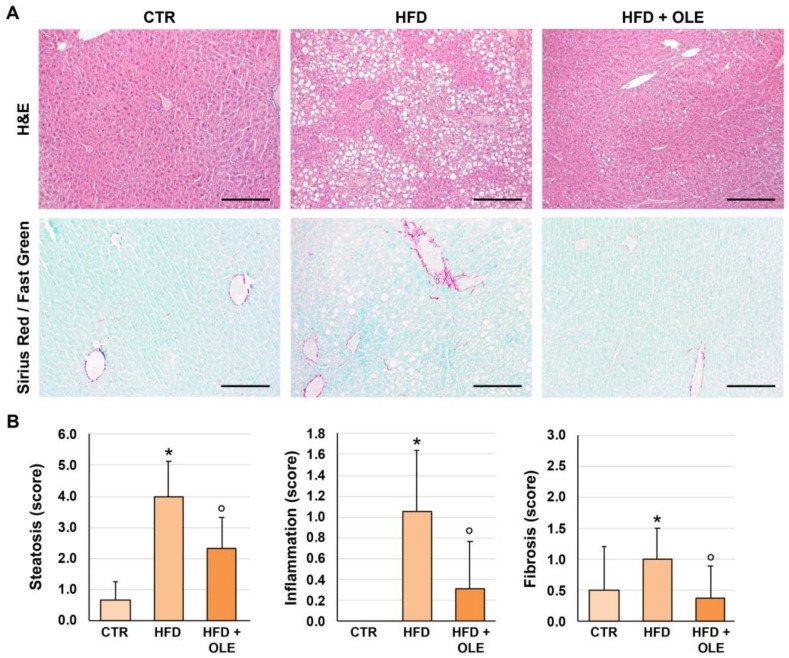
Liver histo-morphology of mice fed a high-fat diet (HFD) and after oleuropein treatment. (**A**) Hematoxylin and eosin (H&E, **upper panels**) and Sirius Red/Fast Green (**lower panels**) stains in the livers of control mice (CTR), HFD mice, and HFD mice treated with oleuropein (HFD + OLE). HFD mice were characterized by higher steatosis, inflammation and fibrosis scores compared to CTR mice. Oleuropein administration was associated with lower steatosis, inflammation, and fibrosis scores compared to untreated HFD mice. Original magnification: 10×. Scale Bar = 200 µm. (**B**). Histograms show means and standard deviation for semi-quantitative scores of liver histo-morphological injury. * *p* < 0.05 vs. CTR; ° *p* < 0.05 vs. HFD.

Histological examinations (Figure 2) showed that HFD mice were characterized by the appearance of significant steatosis and hepatocyte hypertrophy (score = 4.0 ± 1.1), lobular inflammation (score = 1.1 ± 0.6), and fibrosis (score = 1.0 ± 0.5) compared to the controls, in which none of these features could be observed (*p* < 0.001). In the HFD + OLE-treated mice, lower amounts of steatosis (score = 2.3 ± 1.0; *p* = 0.005), lobular inflammation (score = 0.4 ± 0.4; *p* = 0.018), and fibrosis (score = 0.4 ± 0.5; *p* = 0.032) were detected compared to the HFD group.

### 2.3. Oleuropein Reduces HFD-Induced Hepatic LPS Localization and TLR4+ Macrophage Increase

**Figure 3:**
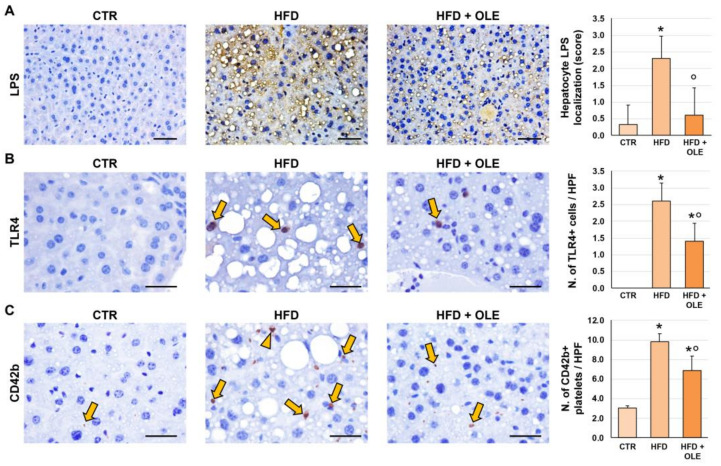
Liver phenotype of mice fed a high-fat diet (HFD) and after oleuropein treatment. (**A**–**C**) Immunohistochemistry for LPS (**A**), TLR4 (**B**), and CD42b (**C**) in the livers of control mice (CTR), HFD mice, and HFD mice treated with oleuropein (HFD + OLE). HFD administration determined a higher hepatocyte LPS localization, and a higher number of TLR4+ macrophages (arrows in (**B**)) and platelets or platelet aggregates (arrows and arrowhead in (**C**)) compared to CTR. Oleuropein treatment was associated with a lower hepatocyte LPS localization, and with a lower number of TLR4+ cells and platelets compared to HFD. Original magnification: 20× (LPS) and 40× (TLR4 and CD42b). Histograms show means and standard deviation for the semi-quantitative score for hepatocyte LPS localization, and for the number of TLR4+ cells and CD42b+ platelets per high-powered field (HPF). * *p* < 0.05 vs. CTR; ° *p* < 0.05 vs. HFD. Scale Bars = 200 µm (**A**) and 25 µm (**B**,**C**).

LPS localization in hepatocytes was quantified by immunohistochemistry on liver sections (Figure 3A). Hepatocyte LPS localization was higher in the HFD mice (score = 2.3 ± 0.7) compared to the controls (score = 0.3 ± 0.6; *p* = 0.006); in the HFD + OLE-treated mice, a lower amount of hepatocyte LPS localization was detected (score = 0.6 ± 0.8) compared to the untreated HFD ones (*p* = 0.007). The HFD mice showed a higher number of TLR4+ macrophages per HPF (2.6 ± 0.5) compared to the controls (negative; *p* < 0.001); in the HFD + OLE-treated mice, a lower TLR4+ macrophage number (1.4 ± 0.5) was revealed compared to the untreated HFD ones (*p* = 0.009) (Figure 3B).

### 2.4. Oleuropein Reduces HFD-Induced Hepatic CD42b+ Platelet Increase

The number of platelets in the liver sinusoids was counted by immunohistochemistry for CD42b (Figure 3C). The number of CD42b+ platelets was increased in liver sinusoids of the HFD mice (9.8 ± 0.8) compared to the controls (3.0 ± 0.3; *p* < 0.001). In the HFD + OLE-treated mice, a lower number of CD42b+ platelets were present (6.9 ± 1.5) in the liver sinusoids compared to the untreated HFD mice (*p* = 0.005).

### 2.5. Oleuropein Reduces HFD-Induced Intestinal Morphological Derangements

**Figure 4:**
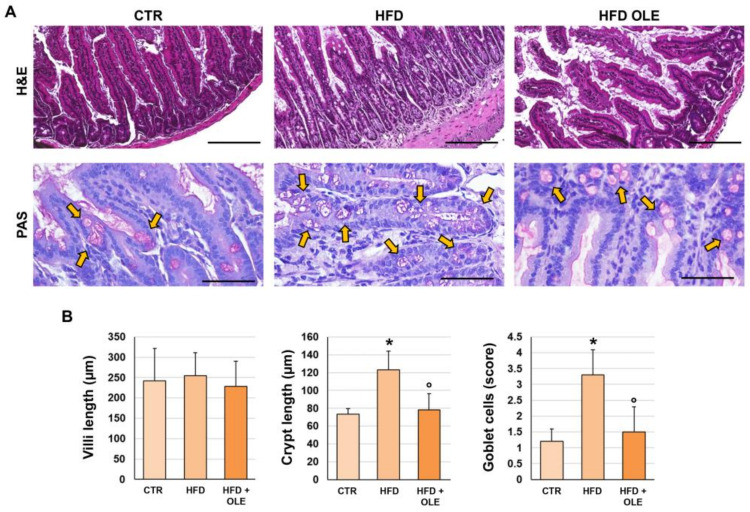
Intestinal histo-morphology of mice fed a high-fat diet (HFD) and after oleuropein treatment. (**A**) Hematoxylin and eosin (H&E, **upper panels**) and periodic acid-Schiff (PAS, **lower panels**) stain in the terminal ileum of control mice (CTR), HFD mice, and HFD mice treated with oleuropein (HFD + OLE). HFD mice showed higher crypt length and goblet cell (arrows) numbers compared to CTR. Oleuropein administration was associated with lower crypt length and goblet cell number compared to untreated HFD. No significant modifications were observed in terms of villi length in HFD and HFD + OLE mice. Scale Bars = 100 µm (**upper panels**) and 50 µm (**lower panels**). (**B**) Histograms show means and standard deviation for villi length, crypt length, and semi-quantitative score of goblet cell number. * *p* < 0.05 vs. CTR; ° *p* < 0.05 vs. HFD.

**Figure 5:**
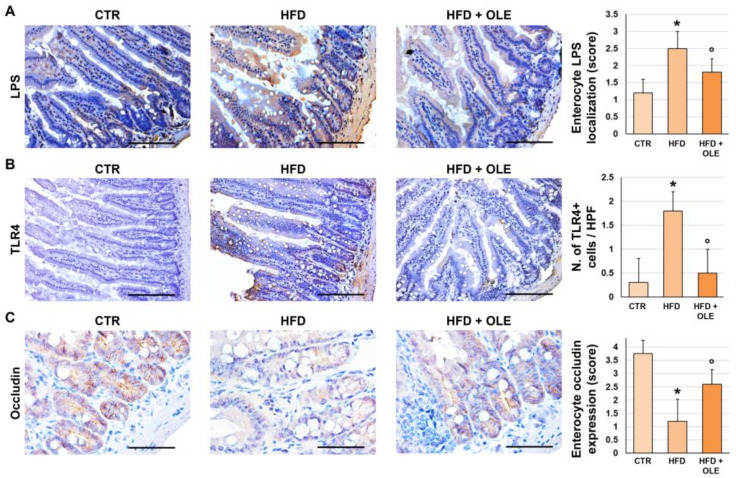
Intestinal phenotypes of mice fed a high-fat diet (HFD) and after oleuropein treatment. (**A**,**B**) Immunohistochemistry for LPS (**A**) and TLR4 (**B**) in the terminal ilea of control mice (CTR), HFD mice, and HFD mice treated with oleuropein (HFD + OLE). HFD administration determined a higher amount of enterocyte LPS localization and a higher number of TLR4+ macrophages compared to CTR. Oleuropein treatment was associated with a lower amount of enterocyte LPS localization and a lower number of TLR4+ cells compared to HFD. Original magnification: 20× (LPS) and 40× (TLR4). (**C**). Immunohistochemistry of occludin in the terminal ileum of control mice, HFD mice and HFD mice treated with oleuropein. HFD administration determined a lower enterocyte occludin compared to CTR. Oleuropein treatment was associated with a higher enterocyte occludin compared to HFD. Original magnification: 40×. In (**A**–**C**), histograms show means and standard deviation for the semi-quantitative score for enterocyte LPS localization, for the number of TLR4+ cells per high-powered field (HPF), and for enterocyte occludin expression. * *p* < 0.05 vs. CTR; ° *p* < 0.05 vs. HFD. Scale Bars = 100 µm (**A**,**B**) and 75 µm (**C**).

LPS translocation induced by HFD could be related to an alteration in the intestine epithelium [19]. Therefore, we evaluated the terminal ilea of the mice in our experimental model (Figure 4 and Figure 5). Intestinal histomorphology was studied on H&E and Periodic acid–Schiff (PAS)-stained slides (Figure 4). A similar length of intestinal villi was observed in the HFD mice (255.0 ± 56.3 µm) and controls (242.1 ± 79.8 µm). However, the HFD mice showed an increased length of intestinal crypts (123.3 ± 20.7 µm) compared to the controls (73.6 ± 6.2 µm; *p* < 0.001), suggesting an activation of the intestinal regenerative compartment. Moreover, the length of the intestinal crypts was lower in the HFD + OLE-treated mice (78.4 ± 18.2 µm) compared to the HFD-fed mice that did not receive OLE (*p* < 0.001).

The percentage of PAS+ goblet cells was higher in the HFD mice (score = 3.3 ± 0.8) compared to the controls (score = 1.2 ± 0.4; *p* < 0.05), indicating a shift in cell differentiation towards a goblet cell fate. Interestingly, in the HFD + OLE-treated mice, an amelioration of this phenotype was observed, as indicated by the lower number of PAS+ goblet cells (score = 1.5 ± 0.8) compared to the untreated HFD mice (*p* = 0.003).

Then, the intestinal expression of LPS and TLR4 was determined by immunohistochemistry (Figure 5). The LPS localization in enterocytes was significantly increased in the HFD mice (score = 2.5 ± 0.5) compared to the controls (score = 1.2 ± 0.4; *p* < 0.001), while a significantly lower amount of enterocyte LPS localization (score = 1.8 ± 0.4) was detected in the HFD + OLE-treated mice compared to the HFD mice (*p* = 0.037). Also, TLR4 expression in enterocytes was significantly increased in the HFD mice (score = 1.8 ± 0.4) compared to controls (score = 0.3 ± 0.5; *p* < 0.001), while a significantly lower TLR4 expression (score = 0.5 ± 0.5) was observed in the HFD + OLE-treated mice compared to the untreated HFD ones (*p* < 0.001).

Lastly, the intestinal positivity for occludin, a tight junction protein, was determined by immunohistochemistry (Figure 5) to evaluate the integrity of the intestinal mucosal barrier. The occludin positivity in enterocytes was significantly reduced in the HFD (score = 1.2 ± 0.8) mice compared to the controls (3.8 ± 0.5; *p* = 0.001); however, the HFD + OLE-treated mice displayed a significantly higher enterocyte occludin positivity (2.6 ± 0.5) compared to the HFD mice (*p* = 0.014).

## 3. Discussion

This study proved that OLE, a component of EVOO, reduces liver inflammation and steatosis by lowering hepatic LPS localization and downregulating intestinal and liver TLR4+ macrophage in HFD-treated mice.

Previous studies provided evidence that liver steatosis induced by HFD is associated with the increased localization of LPS within hepatic cells and the upregulation of TLR4, the receptor of LPS, suggesting a potential cause–effect relationship between LPS and liver inflammation [3,20]. The inflammatory damage elicited by LPS occurs upon its binding to TLR4, which causes the production of inflammatory cytokines that eventually activate inflammation, fibrosis, and the progression of liver damage; in addition, TLR4 knock-out mice are resistant to experimentally-induced NAFLD [2].

Previous studies reported that OLE possesses an anti-inflammatory effect in acute kidney injuries and in ischemia/reperfusion myocardial damage [21,22]. We supported and extended this finding showing that EVOO, which contains OLE, lowers LPS in humans [12,13]; thus, the present study tested the hypothesis that, in animals given an HFD, OLE supplementation could lower the systemic and hepatic levels of LPS, eventually mitigating liver inflammation. Previous studies reported that OLE improved liver steatosis and downregulated hepatic TLR4, but the role of the LPS-TLR4 axis was not investigated [15,23]. We observed that, in animals given an HFD, systemic low-grade endotoxemia, associated with higher levels of inflammatory cytokines, can be detected simultaneously with enhanced LPS localization and TLR4 overexpression in the intestinal tube, suggesting an over-translocation of LPS into systemic circulation. So, we deduced that activating the LPS-TLR4 axis might elicit intestinal inflammation. These changes were counteracted by the chronic administration of OLE, which reduced systemic and intestinal LPS and intestinal TLR4+ macrophages. Together, these data suggest that OLE exerts an anti-inflammatory effect in the intestine. Accordingly, the analysis of intestinal epithelial cells showed a reduced length in the OLE-treated animals compared to the controls, which was paralleled by a lower number of goblet cells in the OLE-treated animals compared to the controls, suggesting that OLE administration mitigated the HFD-induced derangements of the intestinal epithelium. The intrinsic mechanism underlying the translocation of lipopolysaccharide (LPS) into systemic circulation has not been directly investigated yet. We hypothesize that a high-fat diet is linked to an overgrowth of pathogenic bacteria, which adversely affects gut barrier permeability, enabling the translocation of LPS into the portal circulation and, consequently, the amount of LPS reaching liver cells [24]. These changes were counteracted by OLE administration, as suggested by reduced inflammation in the mucosa and the downregulation of the tight junction protein ‘occludin’. Tight junction proteins include transmembrane proteins, e.g., claudins, occludin, tricellulin, and junctional adhesion molecules, and intracellular scaffold proteins, e.g., the zonula occludens proteins ZO-1, ZO-2, and ZO-3, which bind the transmembrane structure to the cytoskeleton [25,26]. In the present study, we observed that OLE administration reduced the circulating levels of zonulin, an indirect marker of improved gut barrier function. Zonulin is a 47 KDa protein released by epithelial cells of the small intestine after stimulation by gliadin or gut dysbiosis [27]. In interstitial epithelial cells, zonulin activation elicits protein kinase C phosphorylation, which triggers the disassembly of tight junction proteins such as ZO-1 [28]. Increased serum levels of zonulin alongside elevated levels of LPS have been detected in patients with type 2 diabetes, individuals with obesity [29,30,31], and patients with acute or chronic cardiovascular disease [32]. Accordingly, our findings indirectly indicate that OLE may positively affect HFD-induced gut dysbiosis-related intestinal barrier permeability, but further investigations are necessary to support this hypothesis. Interestingly, administering EVOO to animals given an HFD counteracted gut dysbiosis with an ensuing production of beneficial metabolites, such as short-chain fatty acids [11].

Parallel changes were detected in the liver of HFD mice that, in fact, displayed inflammatory fingerprints such as LPS accumulation in the liver cells, TLR4 macrophage over-expression, and immunohistochemical evidence of liver inflammation, steatosis, and fibrosis. A beneficial effect of OLE was also observed in the liver compartment, as indicated by a significant reduction in liver inflammation, liver steatosis, and fibrosis along with lowered LPS intrahepatic localization and TLR4 macrophage expression.

Previous studies reported that platelets play a key role in the development of liver steatosis, inflammation, and fibrosis [33,34]. Immunohistochemical studies performed on animals treated with an HFD or in humans affected by NAFLD-NASH documented an in situ increase in platelet cellularity with platelet activation and a tendency for micro-thrombi formation [34]. A cause–effect relationship between platelet activation and liver inflammation/steatosis was documented by a significant reduction in liver inflammation in HFD diet-treated animals given antiplatelet drugs such as aspirin or clopidogrel [3,34]. In the present study, we confirmed that platelets accumulate in the sinusoids of HFD mice and tend to form microaggregates; a significant reduction in platelet number in the liver sinusoids was, however, detected in OLE-treated animals compared to controls, but no changes in micro-aggregates were detected. However, it is noteworthy that systemic P-selectin was reduced in OLE-treated animals, suggesting that OLE exerted an antiplatelet effect; the underlying mechanism, however, needs to be elucidated.

The study has several implications but also some limitations. We provide evidence that, in HFD animals, OLE positively affected intestinal cell homeostasis by reducing intestinal inflammation, improving gut permeability, and ultimately lowering LPS translocation into the systemic circulation, suggesting a positive effect on gut dysbiosis-induced changes in gut permeability typically occurring in animals consuming a high-fat diet [35]; further studies are, therefore, necessary to analyze whether OLE modifies intestinal microbiota or directly affects intestinal cells. LPS can be degraded or blunted at the level of the intestinal tube and liver via enzymes such as alkaline phosphatase or HDL3 [36]; even if we did not observe changes if HDL3 in the systemic circulation, further investigation is necessary to analyze the impact of HFD and HFD + OLE on the pathways modulating intestinal and liver LPS catabolism. The downstream signaling accounting for LPS-TLR4 axis-related liver inflammation was not investigated in the present study and needs to be explored in the future.

## 4. Materials and Methods

### 4.1. Animal Model

We purchased 8–10-week-old C57BL/6 mice from Charles River Laboratories (Margate, UK) and fed them for 8 weeks with either a high-fat diet (HFD) or a control diet [37]. Experiments were approved by the Italian Ministry of Health and by the university’s Commission for Animal Care following the Italian National Research Council (CNR) (No. 13/2024-PR) criteria. Animals received humane care according to the requirements outlined in the Guide for the Care and Use of Laboratory Animals by the National Academy of Sciences and published by the National Institutes of Health.

For the study, the following experimental groups were considered: (i) normally-fed (CTR; n = 15), (ii) ad libitum high-fat diet (HFD)-fed mice (HFD; n = 16), (iii) ad libitum HFD enriched with 1.8 mg/kg oleuropein (HFD + OLE, n = 13), according to our previously clinical trial [38] indicating that 1.8 mg/kg OLE to HFD represents a translationally relevant dose by using standard human–mouse dose conversion tables.

For the HFD model, experiments were performed in 16–18 week-old female and male mice that were fed either a control diet consisting of standard chow (4RF21—by Mucedola) or HFD (60% calories from fat—by Mucedola). The HFD-OLE diet was custom-prepared by the same manufacturer that provided HFD (Mucedola) by adding 1.8 mg/kg of oleuropein (S7867 Selleckem) to their standard HFD recipe. At the end of the experimental period, mice were euthanized by cervical dislocation. Blood samples were drawn by cardiac puncture, and liver and terminal ileum samples were harvested and processed for histology.

### 4.2. HDL3 Assay

The presence of LDH3 in mice serum was detected by commercial immunoassay (MyBioSource, Upper Heyford, UK). The ELISA analytical biochemical technique is based on LDH3 antibody–LDH3 antigen interactions (immunosorbency) and an HRP colorimetric detection system that is used to detect LDH3 antigen targets in samples. The values were expressed as ng/mL, and intra- and inter-assay coefficients of variation were <15%.

### 4.3. Tissue Sampling and Histo-Morphological and Immunohistochemical Stains

The livers and terminal ilea were harvested and fixed in 4% buffered formalin at sacrifice. Samples were embedded in low fusion temperature paraffin, and 3 µm sections were obtained. Routine histological stains were performed, i.e., hematoxylin–eosin (H&E), Sirius Red/Fast Green (SR/FG), and periodic acid–Schiff (PAS) stain. For immunohistochemistry, endogenous peroxidase activity was blocked by a 30 min incubation in methanolic hydrogen peroxide (2.5%). As indicated by the vendor, antigens were retrieved by applying Proteinase K (Dako, Glostrup, Denmark, code S3020) for 10 min at room temperature. Sections were incubated overnight at 4 °C with primary antibodies (Table 1). Then, samples were rinsed twice with phosphate-buffered saline (PBS) for 5 min, incubated for 20 min at room temperature (RT) with secondary biotinylated antibody, and then with Streptavidin–horseradish peroxidase (LSAB+, Dako, Glostrup, Denmark code K0690). Diaminobenzidine (Dako, Glostrup, Denmark code K3468) was used as a substrate, and sections were counterstained with hematoxylin. For all immunoreactions, pre-immune serum was also included as a negative control. Histological sections were analyzed by a photonic microscope, Leica Microsystems DM 4500 B, equipped with a video camera Jenoptik Prog Res C10 Plus, (Leica Biosystems, Milan, Italy) by two blinded independent researchers (sample identity was hidden to them using coded slides). Slides were further scanned by a digital scanner (Aperio ScanScope^®^ CS and FL Systems; Aperio Digital Pathology, Leica Biosystems, Milan, Italy) and processed by ImageScope version 12.3.3).

### 4.4. Histopathology and Immunophenotype of Liver and Intestine

Histopathological evaluation of the liver was performed according to previous indications and included hepatocyte steatosis, hepatocyte hypertrophy, lobular inflammation, and fibrosis [39]. For the intestine, terminal ileum samples were radially cut; mean crypt and villus length were quantitated from ten non-overlapping 20× fields per mouse on H&E-stained slides. Briefly, digital slides were processed by ImageScope software and length was measured by using the ruler tool. The number of goblet cells was counted in 20 randomly selected PAS-stained crypts/villi with sagittal orientation. The goblet cell density was measured by dividing the goblet cell number per field by the total number of cells and is expressed as a semi-quantitative score (see below).

The number of CD42b+ platelets and TLR4+ macrophages was calculated as the number of cells per high-powered field (HPF, i.e., 40× magnification). Platelets were identified as small cellular fragments positive for platelet marker CD42b. Based on previous evidence [3], TLR4+ inflammatory cells in liver sinusoids were considered as activated pro-inflammatory macrophages. For LPS expression, the number of positive cells was automatically calculated by an algorithm on the entire section, and then, a semi-quantitative scoring system was applied (0: ≤1%; 1: 1–10%; 2: 10–30%; 3: 30–50%; 4: ≥50%). For each slide, at least 10 non-overlapping microscopic fields were randomly chosen.

### 4.5. Serum LPS

LPS serum levels in mouse serum were measured using a commercial ELISA kit (Cusabio, Wuhan, China). The samples were plated for 2 h at room temperature onto a microplate pre-coated with the antibody specific for LPS. After incubation, samples were read at 450 nm. Values were expressed as pg/mL; intra-assay and inter-assay coefficients of variation were <10%.

### 4.6. Soluble Platelet Selectin (sP-Selectin) Assay

Levels of soluble P-selectin (sP-selectin), a marker of platelet activation in vivo, were measured with a commercial immunoassay in the sera of mice (DRG International, Springfield, IL, USA). Intra- and inter-assay coefficients of variation were <10%. The sample values were expressed in ng/mL.

### 4.7. Zonulin Assay

Zonulin serum levels were measured using a commercial ELISA kit (Elabscience, Huston, TX, USA). Zonulin-specific antibodies were pre-coated onto a microplate and were incubated for 90 min at 37 °C with patients’ sera samples. Then, a biotinylated anti-zonulin antibody and avidin–horseradish peroxidase (HRP) conjugate were added to each microplate. The amount of zonulin was measured with a microplate auto-reader at 450 nm. Values were expressed as ng/mL; both intra-assay and inter-assay coefficients of variation were <10%.

### 4.8. Interferon γ (IFN-γ) Assay

The concentrations of IFN-γ were quantified using a commercial ELISA kit (Abcam, Cambridge, UK) according to the manufacturer’s instructions. Values were expressed as pg/mL; both intra-assay and inter-assay coefficients of variation were <10%.

### 4.9. Tumor Necrosis Factor-α (TNF-α) Assay

TNF*α* levels were evaluated in samples by a commercial immunoassay kit (Abcam, Cambridge, UK), and values were expressed as pg/mL; intra- and inter-assay coefficients of variation were <10%.

### 4.10. Statistical Analysis

Continuous variables were expressed as means ± standard deviation. Mann–Whitney U-test was used to study differences among groups. All tests were two-tailed, and the statistical significance was set at a *p*-value of less than 0.05. Analyses were performed using computer software packages (IBM SPSS Statistics v25, Armonk, NY, USA).

## 5. Conclusions

The present study shows that in animals given an HFD in conjunction with the administration of OLE results in lowered LPS localization in the intestinal tube and hepatocytes, and downregulation of TLR4, with ensuing reduced liver inflammation and steato-fibrosis. This finding warrants further study to assess if OLE administration may represent a novel approach to attenuate NAFLD and its sequelae in human patients.

## Figures and Tables

**Table 1:** List of primary antibodies.

Antibody	Host Species	Manufacturer	Code	Dilution	Application
*E. coli* LPS	Mouse	abcam	ab35654	1:50	IHC
CD42b	Rabbit	abcam	ab183345	1:100	IHC
TLR4	Rabbit	Atlas Antibodies	HPA049174	1:200	IHC
Occludin	Rabbit	abcam	ab216327	1:100	IF

## Data Availability

Data will be made available to the corresponding author upon request.
